# Pelota-interacting G protein Hbs1 is required for spermatogenesis in *Drosophila*

**DOI:** 10.1038/s41598-019-39530-6

**Published:** 2019-03-01

**Authors:** Zhaohui Li, Fu Yang, Yang Xuan, Rongwen Xi, Rui Zhao

**Affiliations:** 1 School of Life Sciences, Tsinghua University, Beijing, 100084 China; 2 National Institute of Biological Sciences, No. 7 Science Park Road, Zhongguancun Life Science Park, Beijing, 102206 China; 3 Tsinghua Institute of Multidisciplinary Biomedical Research, Tsinghua University, Beijing, China; 4 Present Address: Genomics Institute of the Novartis Research Foundation, San Diego, California 92121 USA

## Abstract

Hbs1, which is homologous to the GTPase eRF3, is a small G protein implicated in mRNA quality control. It interacts with a translation-release factor 1-like protein Dom34/Pelota to direct decay of mRNAs with ribosomal stalls. Although both proteins are evolutionarily conserved in eukaryotes, the biological function of Hbs1 in multicellular organisms is yet to be characterized. In *Drosophila*, *pelota* is essential for the progression through meiosis during spermatogenesis and germline stem cell maintenance. Here we show that homozygous *Hbs1* mutant flies are viable, female-fertile, but male-sterile, which is due to defects in meiosis and spermatid individualization, phenotypes that are also observed in *pelota* hypomorphic mutants. In contrast, *Hbs1* mutants have no obvious defects in germline stem cell maintenance. We show that *Hbs1* genetically interacts with *pelota* during spermatid individualization. Furthermore, Pelota with a point mutation on the putative Hbs1-binding site cannot substitute the wild type protein for normal spermatogenesis. These data suggest that Pelota forms a complex with Hbs1 to regulate multiple processes during spermatogenesis. Our results reveal a specific requirement of Hbs1 in male gametogenesis in *Drosophila* and indicate an essential role for the RNA surveillance complex Pelota-Hbs1 in spermatogenesis, a function that could be conserved in mammals.

## Introduction

Spermatogenesis in *Drosophila* is initiated by the asymmetric divisions of germline stem cells (GSCs) that give rise to the respective differentiated gonialblasts, each of which then undergoes four rounds of mitotic divisions to produce 16 spermatocytes. The spermatocytes then go through two rounds of meioses to yield a cyst of 64 spermatids. At the late stage of spermatogenesis, spermatids undergo morphological changes, such as elongation and individualization, where 64 syncytial spermatids are separated into 64 individual mature sperms^1^. During meiosis and spermiogenesis, gene transcription is globally reduced and the transcripts used for late stages are stored in the cytoplasm until translation. Therefore, gene regulation at the translational level plays an important part to ensure proper sperm development.

*Pelota (pelo)*, which encodes an evolutionarily conserved eukaryotic release factor 1-like protein, was first identified in a genetic screen for genes involved in male fertility in *Drosophila melanogaster*^2^. Mutation in the *pelo* locus causes cell cycle arrest at G2/M transition before the first meiotic division during spermatogenesis^3^. Deletion of *dom34*, the orthologue of *pelo* in yeast, results in defective sporulation and decreased polyribosomes, suggesting that the molecular function of Pelo is related to translational regulation^4^. Dom34 was found to interact with Hbs1, originally uncovered in yeast as a suppressor of the growth defect in strains deficient for Hsp 70 proteins^5^, and Hbs1 was also shown to promotes efficient cell growth and protein synthesis^6^. Importantly, both Dom34p and Hbs1 are identified as important factors in a mRNA quality control pathway named no-go decay, in which mRNAs with ribosomal stalls during translational elongation or at the 3′ end of mRNA are subjected to degradation by endonucleolytic cleavage^7–10^. The role of Pelo in mRNA quality control is also confirmed in *Drosophila* and human cells^8,11–13^.

Structural analysis of archaeal and yeast homologues of Pelo suggests that it is related to eukaryotic release factor 1 (eRF1) in the central and the C-terminal domains except some differences in the N-terminal region, but does not contain the stop codon recognition motif or GGQ motif that helps to catalyze hydrolysis of peptide^14,15^. eRF1 shares a three-dimensional structure similar to tRNA and functions to terminate translation by recognition of the stop codon at A site of ribosome and hydrolysis of the peptidyl-tRNA bond for peptide release^16^. eRF1 binds to eRF3 through its C-terminal domain and this interaction increases the affinity of eRF3 for GTP^17^. Similarly, Pelo-Hbs1 interaction is able to augment the affinity constant of Hbs1 to GTP^14^. Structure of the Dom34-Hbs1 complex confirms the analogy of the overall similarity to that of eRF1-eRF3, but also reveals that the N-terminal region of Pelo may function differently^18–20^. The binding of Hbs1 to Dom34 leads to a conformational change with a tRNA-like structure formation by the middle domain of Dom34 and positioning a conserved basic region expected for translational termination. Indeed, Dom34-Hbs1 shows maximum kinetic efficiency on the ribosome complexes that contain truncated mRNAs^21^ and promotes dissociation of stalled elongation complexes and release of peptidyl-tRNA in an A site codon-independent manner^10^. Additionally, the Dom34/Hbs1/Rli1 complex mediates the dissociation of the stalled ribosomes into 40 S and 60 S subunits. Taken together, the Pelo-Hbs1 complex regulates gene expression at translational level by facilitating the recycle of ribosomes and removal of aberrant mRNAs when elongation stalls occur.

The Pelo-Hbs1 complex seems to be conserved throughout evolution. Although Hbs1 is absent in archaea, its function is performed by elongation factor aEF1a^22,23^. In mammals, Pelo-Hbs1 also promotes dissociation of elongation complexes, but only with an ABC-type ATPase ABCE1 in the complex^12,24,25^. Also, mammalian Pelo seems to have additional roles outside of mRNA quality control and the pleiotropic function of Pelo might be also reflected by the observation that *pelo* null mice die as early as embryonic day 7.5^12,26^. Conditional knock-out of *Pelo* at postnatal stages revealed that *Pelo* is essential for the maintenance of spermatogonial stem cells by regulating PI3K/AKT pathway and the downstream FOXO1 activity^27^. In *Drosophila*, mutation in *pelo* also causes loss of ovarian GSCs, in part as a result of compromised BMP signaling pathway activation^28^. These observations indicate an evolutionarily conserved function of Pelo in GSCs and gonad development. We have previously shown that Pelo-Hbs1 is required for transposon silencing in the female germline^29^. However, the biological function of Hbs1 and Pelo-Hbs1 complex beyond female germline remains to be explored in multicellular organisms.

Here in this study, we investigated the biological function of *Drosophila* Hbs1 by generating and analyzing *Hbs1* mutants. We find that *Hbs1* mutants are male sterile due to defects in meiosis and spermatid individualization during spermatogenesis. We show that Pelo is also required for spermatid individualization in addition to its known role in early meiosis during spermatogenesis, revealing that Pelo functions at multiple stages during male germline development. By interaction analysis, we show that Hbs1 and Pelo genetically and physically interact with each other. We thus demonstrate an essential role for the Pelo-Hbs1 complex in spermatogenesis, likely through translational regulation.

## Results

### *Hbs1* is required for male fertility in *Drosophila*

**Figure 1:**
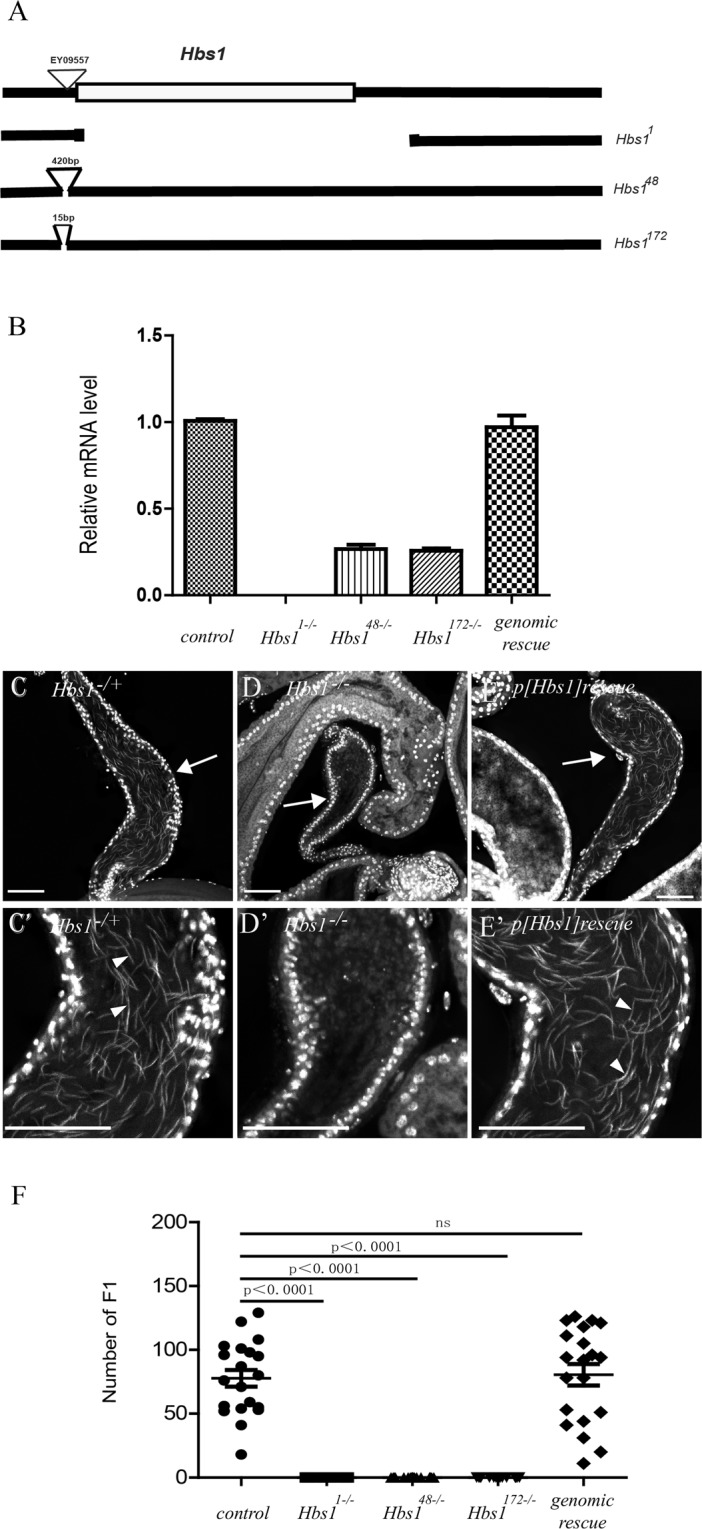
*Hbs1* mutants are male sterile. (**A**) A scheme shows the genomic region of *Hbs1* gene and the respective molecular lesions of the three alleles, *Hbs1*^*1*^, *Hbs1*^*48*^ and *Hbs1*^*172*^. In *Hbs1*^*1*^ allele, the entire coding region of *Hbs1* was removed by imprecise excision of the P element EY09557 inserted before the transcriptional initiation site. In *Hbs1*^*48−/−*^ and *Hbs1*^*172−/−*^ alleles, there were additional 420-bp and 15-bp DNA fragments, respectively, left by the P element. (**B**) Quantitative RT-PCR analysis of the relative *Hbs1* expression in the three *Hbs1* mutants and genomic rescue flies. Hbs1 expression could hardly be detected in *Hbs1*^*1−/−*^ testis and was significantly down-regulated in *Hbs1*^*48−/−*^ and *Hbs1*^*172−/−*^ alleles. The transgenic flies carrying the genomic region of *Hbs1* could restore the expression of *Hbs1* in mutant animals to the normal level. Mean ± SEM, *n* = 3. (**C**) Seminal vesicles (sv, arrow) of *Hbs1*^*1*^ heterozygotes were full of sperm nuclei(arrow head). (**D**) No sperm was detected in *Hbs1*^*1−/−*^ mutant seminal vesicle, and the sv was smaller than that of the *Hbs1*^*1*^ heterozygote. (**E**) The spermless phenotype could be fully rescued by a transgene carrying the genomic region of *Hbs1*. (**F**) A graph showing the fertility analysis of the *Hbs1* mutants and genomic rescued flies. *Hbs1* mutant males were sterile. The sterility could be rescued by the introduction of genomic *Hbs1*. Mean ± SEM. *n* = 20. *p* < *0*.*0001*, *t test*. In images (**C–E**), samples were stained with the DNA dye DAPI (white). Scale bars = 50 μm.

ORF of *Hbs1* (CG1898) in *Drosophila melanogaster* encodes a polypeptide of 670 amino acids, with a predicated molecular weight of approximately 74 kDa. Hbs1 is a highly conserved protein in eukaryotic kingdoms, with the full length of fly counterpart sharing a similarity of 48%, 37%, 48%, 45% and 45% with the homologs in yeast, C. elegans, Xenopus, chicken and human, respectively (Fig. S1). *Drosophila* homologue of Hbs1 contains two conserved domains, a 240 amino acid N-terminal domain of unknown function and a 430 amino acid C-terminal domain bearing four GTP binding sites. *Hbs1* is located at the cytological position 62B on the left arm of chromosome 3. To study its biological function, we conducted P element-mediated excision from a P-element insertion allele *Hbs1*^EY09557^ to generate *Hbs1* mutants. Via PCR screens, we identified three deletion or insertion alleles among 400 excised lines. One allele, named *Hbs1*^*1*^, has a 2307 bp deletion (spans from 1863069 to 1865375 on chromosome 3 L) that covers the entire coding region of *Hbs1* (Fig. 1A), and is therefore a null allele. Two other alleles, *Hbs1*^*48*^ and *Hbs1*^*172*^, has a 420 bp and a 15 bp insertion, respectively, left by the original P-element (Fig. 1A). RT-PCR analysis revealed that the *Hbs1*^*1*^ null allele led to the absence of *Hbs1* expression, while the *Hbs1*^*48*^ and *Hbs1*^*172*^ alleles showed down-regulation of *Hbs1* expression (Fig. 1B). All *Hbs1* mutants were homozygous viable. Moreover, *Hbs1*^*1*^ homozygous females were fertile and produced normal offspring, ruling out a possible maternal function of *Hbs1* for viability. However, for all three alleles, homozygous mutant males were sterile (Fig. 1F). Mutant testes looked morphologically normal. However, there was no sperm found in seminal vesicles (SV) and mutant SVs were much smaller than that in control flies. (Fig. 1C,D), suggesting that no mature sperm is formed in the absence of *Hbs1*. This sterility was efficiently rescued by a transgene containing the genomic region of *Hbs1* (Fig. 1E,F), demonstrating that *Hbs1* mutation is solely responsible for the male-sterile phenotype.

### *Hbs1* mutants are defective at meiosis and spermatid individualization during spermatogenesis

**Figure 2:**
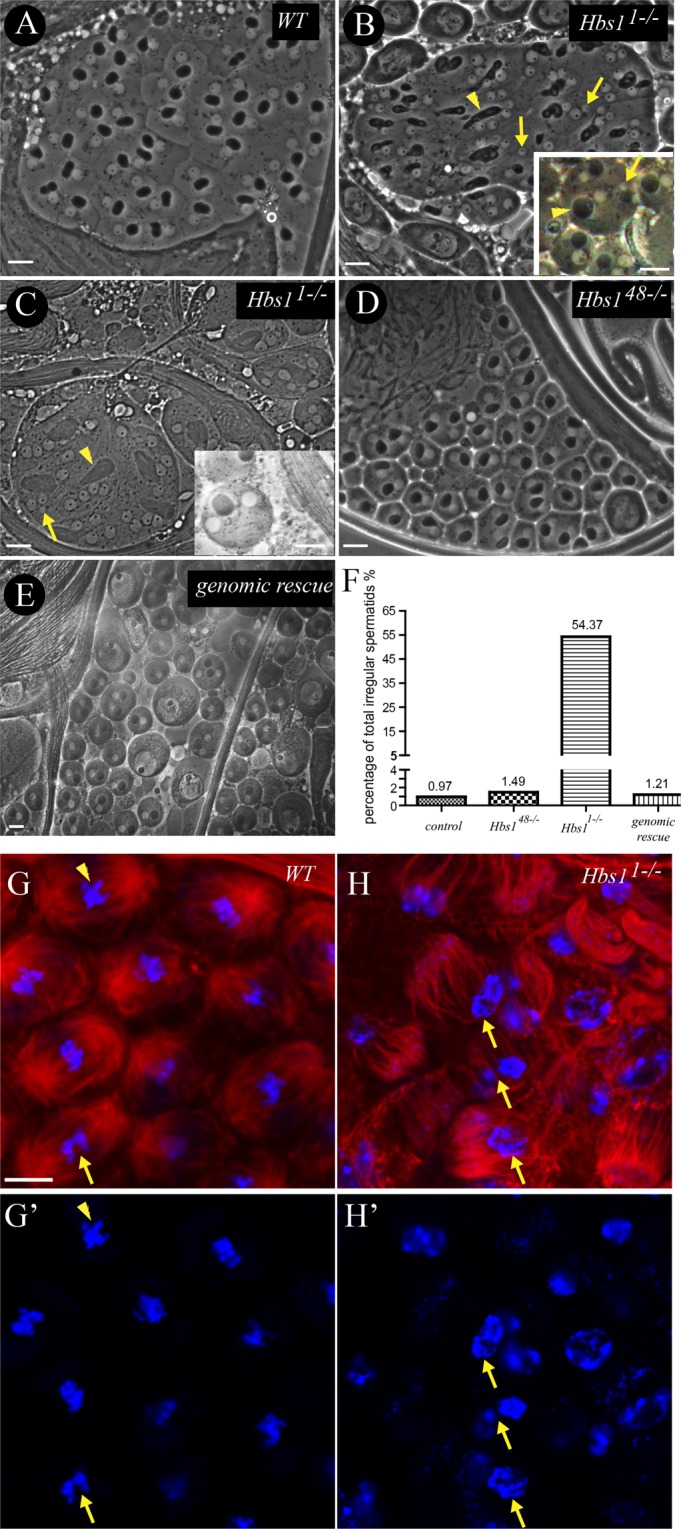
*Hbs1* is required for male meiosis. (**A**) In wild-type cysts, each spermatid contained one nebenkern (dark) to one nucleus (light) (1:1 in ratio) of similar sizes. (**B**,**C**) In some of the *Hbs1*^*1−/−*^ mutant cysts, heterogeneous mixture of ratios of nebenkern to nucleus (from 1:1, 1:2, 1:3 to 1:4) and abnormal large nebenkern(arrow heads) with small or micronuclei(arrows) were frequently observed, which was evidence of failure of cytokinesis and chromosome mis-segregation (See Table 1 for the quantitative data). (**D**) The meiotic figure appeared normal in *Hbs1*^*48−/−*^ mutant cysts. (**E**) Normal onion stage spermatids were observed in the Hbs1 genomic rescued testis. (**F**) There was greater percentage of total irregular spermatids in the *Hbs1*^*1−/−*^ testes. (**G**,**H**) The wildtype testis (**G**–**G’**) exhibited fully formed and well-focused spindle poles, and the condensed chromosomes were either at the metaphase plate (arrowhead) or beginning to separate (arrow). In *Hbs1*^*1−/−*^ testes, the spindle microtubules failed to converge into the poles and were splayed outward, and the chromosome could not be correctly separated (arrow).α-tubulin staining is in red, and DAPI is in blue. Scale bars = 10 μm.

We next investigated the underlying mechanisms of spermatogenesis failure caused by the loss of *Hbs1*. Meiotic figures with one dark nebenkern and one light nucleus of similar size, which are the results of correct meiotic divisions, could be observed in the 64 connected spermatids of squashed wild-type testis by phase contrast microscopy (Fig. 2A), as previously described^1^. Correct meiotic divisions depend on the accurate chromosome segregation and cytokinesis. Arrest of cytokinesis during the first or second division in meiosis results in spermatids with two or four normal size nuclei associated with an abnormally large nebenkern. Nuclear size is proportional to its chromosome content in the onion-stage spermatids^1^. Unsuccessful chromosome segregation results in spermatids with abnormal numbers of nuclei of abnormal size, even with micronuclei^30,31^. Failure in both cytokinesis and chromosome segregation together produces spermatids with large nebenkern associated with multiple nuclei of different sizes^32,33^. Spermatogonia and spermatocytes from *Hbs1*^*1*^ testis showed comparable sizes to the wild type counterpart but abnormal nebenkern to nucleus ratio of 1:2, 1:3 and 1:4 (Fig. 2B,C, Table 1). Irregular size of nebenkerns and nuclei in the spermatids was frequently observed in mutant testes (Fig. 2B,C,F, Table 1), suggesting the presence of a defect in *Hbs1*^*1*^ germline cysts to properly undergo cytokinesis and progress through meiosis. The spindle checkpoint is not stringent in spermatocytes and causes only a small delay in meiotic progression^31,33–35^, which is revealed by the presence of only 22.1% spermatids containing nuclei of abnormal size, as shown in Table 1. The meiosis defect was rescued by the transgene carrying the genomic region of *Hbs1* (Fig. 2E,F. Table 1), confirming that *Hbs1* is required for meiosis during spermatogenesis. In addition, we stained for DNA and α-tubulin to directly examine the spindle formation during meiotic division in wild type and Hbs1 mutant testes. We frequently found that the spindle microtubules failed to converge into the poles and were splayed outward in *Hbs1*^*1−/−*^ testes and consequently the chromosome could not be correctly separated (Fig. 2G,H’). These observations provide an explanation to the initially observed abnormal-sized nuclei phenotype found in *Hbs1*^*1−/−*^ testes.

**Table 1:** Abnormal Spermatid Morphology in *wt* and *Hbs1* mutants.

genotype	total spermatid number	number of different spermatid types							
		1:1		1:2		1:4		other irregular	total irregular
		regular	irregular	regular	irregular	regular	irregular		
*wt*	619	613	1	3	0	0	0	2	6
*Hbs1* ^*48*^ */Hbs1* ^*48*^	404	398	0	2	0	1	0	3	6
*Hbs1* ^*1*^ */Hbs1* ^*1*^	412	188	27	87	53	24	11	22	224
genomic rescue	578	571	2	5	0	0	0	0	7

Ratios refer to the number of nebenkerns to the number of nuclei. “Regular” means the nuclei are of similar size (Fig. 2C insert). “Irregular” means there are abnormal size of nuclei (Fig. 2B insert). “Other irregular” are irregular spermatids that are not included the former 6 kinds, such as one regular nebenkern with 3 nuclei of different size, abnormal size nebenkerns, and so on. The number of “total irregular” is the sum of all the abnormal spermatids, including the spermatids with the abnormal size nuclei (the “irregular”) and the spermatids with abnormal ratio of nebenkerns to nuclei (1:2, 1:4 and others irregular).

Interestingly, in *Hbs1*^*48*^ (Fig. 2D,F) or *Hbs1*^*172*^ homozygous mutant testes, the abnormal germline cysts found in the null mutants were not observed. But still, *Hbs1*^*48*^ and *Hbs1*^*172*^ homozygous testes contained no sperm in the seminal vesicles. Moreover, there were also some cysts with normal meiotic figures in *Hbs1*^*1*^ testes, yet no sperm was detected in the mutant seminal vesicles, suggesting that *Hbs1* is also required during later spermatogenesis.

**Figure 3:**
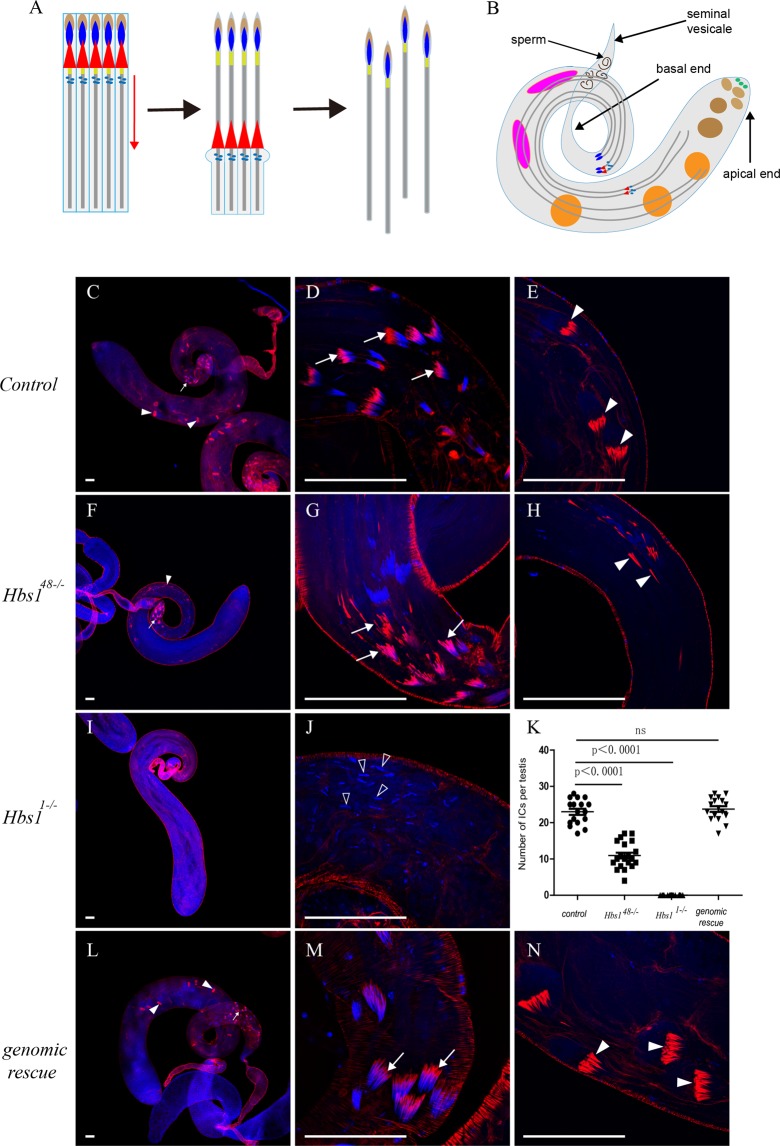
*Hbs1* is required for spermatid individualization. (**A**) A schematic representation of four spermatids undergoing individualization: actin cones travel along the spermatid axonemes from the heads (the nuclear side) to the tips of the tails during the individualization. The cytoplasmic content is removed and the syncytial membrane is remodeled during the movement of actin cones. The syncytial spermatids (far left) are finally divided into individual sperm(far right). Dark blue: spermatid nuclear bundle, red: actin cone, light blue: Golgi, light brown: acrosome; yellow: basal body, grey: spermatid axoneme. (**B**) A schematic diagram of the fly testis. light green: stem cell, light brown: spermatogonia, dark brown: spermatocytes, orange: spermatids, pink: beginning of spermatids elongation, purple: elongated spermatids of different individualization stage (dark blue: spermatid nuclear bundle, red: actin cone, light blue: Golgi). (**C**–**E**) Intact individualization complexes (ICs, stained with phalloidin in red) were detected in wild type controls and 64 intact nuclei (revealed by DAPI staining in blue) were clustered together. ICs moved from the basal end(**C**,**D**) towards apical end (**C**,**E**). (**F**–**H**) In *Hbs1*^*48−/−*^ testes, ICs were formed but they were slightly disorganized (**F**,**G**) and they could not properly move away with disorganized actin cones scattering along the testicular tube (**F**,**H**). (**I**,**J**) ICs could not be detected in *Hbs1*^*1−/−*^ mutant testis, and spermatid nuclei were randomly scattered along the testicular tubes (indicated by hollow arrowheads). (**K**) The number of ICs in *Hbs1*^*48−/−*^ and *Hbs1*^*1−/−*^ mutant testis was less than that in wild type control testes. ICs number was restored in the flies with the genomic rescue of Hbs1. Mean ± SEM. *n* = 17 for control and genomic rescue testes, n = 20 for *Hbs1*^*48−/−*^ and *Hbs1*^*1−/−*^ testes, *p* < *0*.*0001*, *t test*. (**L**,**N**) Introduction of the genomic construct of *Hbs1* in mutant flies rescued the nuclear bundling and subsequent ICs formation defect and ICs properly progressed away. The ICs near the basal end, which is adjacent to the nuclei, are indicated by the arrows. The ICs (actin cones) near the apical end are indicated by arrow heads. Scale bars = 50 μm.

Individualization is the final stage of spermatogenesis, during which the syncytial membrane is remodeled and a whole cyst of 64 syncytial spermatids is divided into 64 individual sperm (Fig. 3A)^36,37^. Individualization complex (IC), which consists of 64 actin cones, is required for this process. At the beginning of individualization, the actin cones assemble around the spermatid nuclei towards the basal end of the testis (Fig. 3A,B,D), and then move away from the nuclei along the spermatid axonemes to the apical end of the testis (Fig. 3A,B,E). In wild type testes, there were spermatids at different individualization stages with about 23 intact ICs (Fig. 3K) that scattered along the testicular tube from basal end (the initial stage of individualization, Fig. 3B–D) to apical end (the late stage of individualization, Fig. 3B–C,E). Each actin cone could be visualized by staining for rodamine-phalloidin^36^. At the initial stage of individualization, ICs were adjacent to spermatid nuclei as revealed by positive phalloidin signals immediately next to DAPI staining (Fig. 3C,D). At the late stage of individualization, ICs were far away from the nuclei (Fig. 3C,E). In *Hbs1*^*48*^ (Fig. 3D–F) and *Hbs1*^*172*^ homozygotes, there were only about 11 disorganized ICs (Fig. 3F–G,K) near the basal end. No intact IC near the apical end was observed and only dissociated actin cones scattering along the testicular tube were detected (Fig. 3F,H), which indicates that ICs could not properly progress away from the nuclei. ICs were basically absent in *Hbs1*^*1*^ homozygous testes and all the spermatid nuclei randomly scattered along the testicular tube (Fig. 3I–K), suggesting a block in nuclear bundling, and consequently, a defect in IC formation in *Hbs1* null testis. These defects were rescued by the transgene carrying genomic *Hbs1* (Fig. 3K–N). Together, these observations suggest that *Hbs1* is required for proper nuclear bundling and proper traveling of ICs during the individualization stage of spermiogenesis.

### *Hbs1* genetically interacts with *pelo* during spermatogenesis

Since both *pelo* and *Hbs1* mutants in *Drosophila* showed defects in meiosis during spermatogenesis, we asked whether Pelo and Hbs1 could function together to regulate spermatogenesis. The existing allele of *pelo*, namely *pelo*^1^, is a P-element-insertional allele, which produces a truncated transcript of the gene. Mutant fly homozygous for *pelo*^1^ has an early cell cycle arrest at the first meiotic cell division during spermatogenesis^3^. *Hbs1* mutants apparently affect both meiosis and individualization during spermatogenesis, but it remains unknown whether *pelo* also plays a role in spermiogenesis besides its function in meiosis. We therefore generated several new *pelo* alleles by imprecise P-element-mediated excision^29^. We classified these alleles from weak to strong according to the severity of phenotypes and molecular lesions, as described in Materials and Methods.

**Figure 4:**
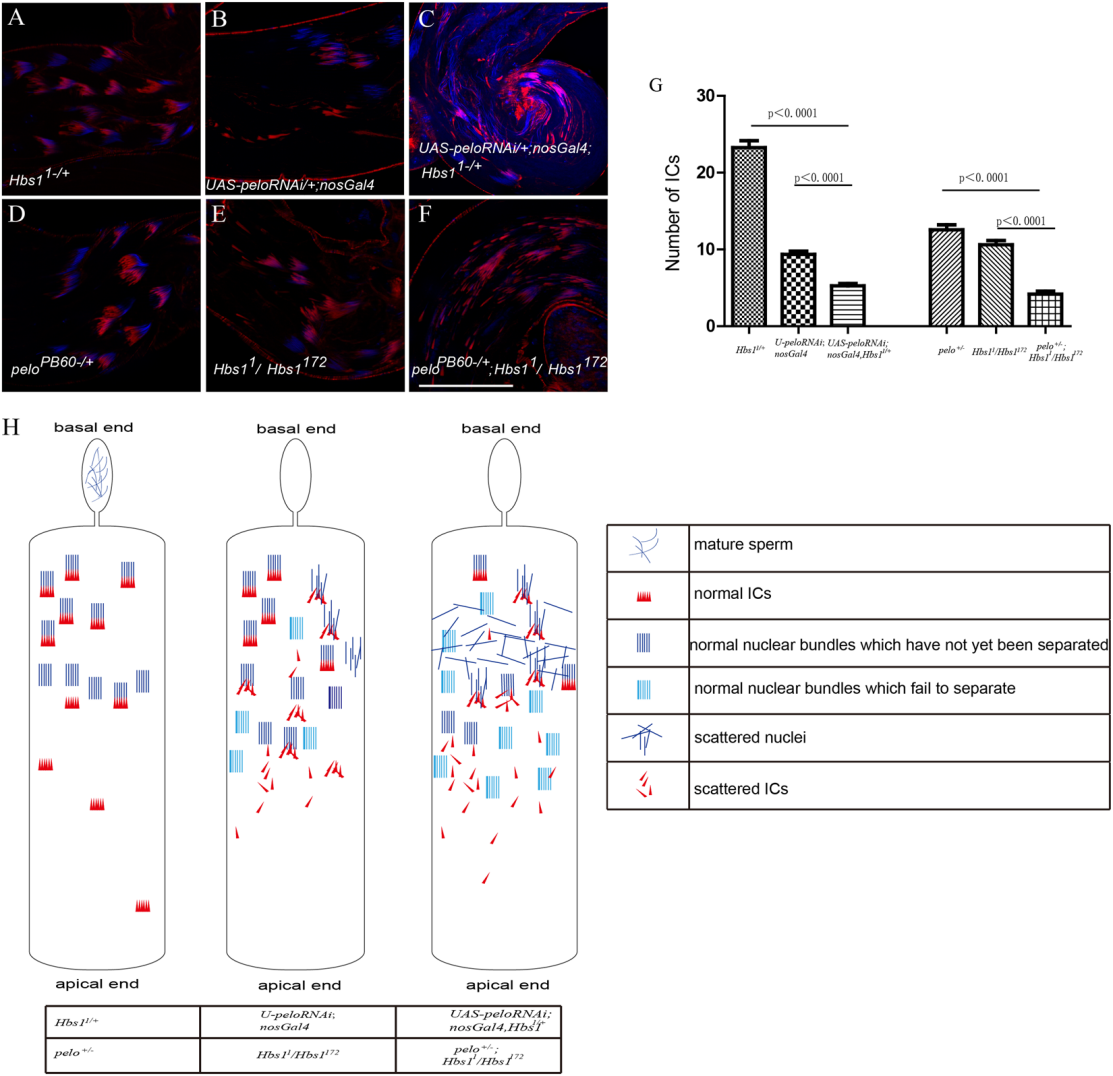
Genetic interaction between *Pelo* and *Hbs1* during spermatogenesis. (**A**) Removal of one copy of *Hbs1* in wild-type background displayed no discernible phenotype on IC assembling. (**B**) Scattered ICs and nuclei were frequently detected in testes genotyped UAS-*pelo*RNAi/+; nosGal4/+. (**C**) *Pelo-*RNAi with one copy of *Hbs1* removed led to an increase in severely disorganized nuclei and a dramatic inhibition in proper IC assembling(**C**). (**D**) Testes heterozygous for *pelo*^*PB60*^ displayed normal spermatid and IC morphology. (**E**) *Hbs1* mutant testes with *Hbs1*^*1*^ in trans to *Hbs1*^*172*^ displayed mild IC disorganization phenotype. (**F**) Loss of one copy of *pelo* in *Hbs1*^*1*^*/Hbs1*^*172*^ trans-heterozygotes led to decreased number of ICs and more disorganized nuclei and ICs. Comparisons of IC number per testis among different genetic background are summarized in (**G**) (the normal looking ICs were quantified, including the ICs that had progressed down in apical end.). (**H**) Schematic representation of the degree of the nuclear and IC defects in these flies. IC disorganization and nuclei scattering phenotype was more severe in the *pelo*^*PB60*^/+; *Hbs1*^1^*/Hbs1*^*172*^ and UAS-*pelo*RNAi/+; nosGal4/+; *Hbs1*^*1*^/+ flies and the nuclear bundles, which failed to separate, accumulated in the testis tube. Mean ± SEM. n = 20. p < 0.0001, t test. Scale bars = 50 μm.

Meiotic defects are generally more severe in *pelo* mutants than that in *Hbs1* mutants. Strong loss-of-function (LOF) *pelo* mutants like *pelo*^*PB60*^ and *pelo*^1^ showed a total blockage of cell division, and mutant spermatocytes did not enter meiosis. Weak *pelo*^*PA13*^ mutants displayed cell division arrest either before or at the first meiotic division (Fig. S2A–F), as previously reported^3^. Despite the observations that the 2N and 4N spermatids seemed to be able to initiate nuclei elongation in the mutants, there was no IC formation in these *pelo* mutant testes (Fig. S2G,H). Knocking down *pelo* by two RNAi lines, however, generated weaker phenotypes such that spermatocytes went through meiosis, but ICs showed disorganization (Fig. 4A,G) similar to hypomorphic *Hbs1* mutants (Fig. 3H).

We then analyzed potential genetic interactions between *Hbs1* and *pelo* during spermatid individualization. We found that *pelo*-RNAi in the germline caused defects in the organization of ICs (Fig. 4B,H) and there were only about a number of 9.4 relatively normal-sized ICs in these testes (Fig. 4G). However, IC number declined significantly to an average of 5.3 when one copy of *Hbs1* was further removed (Fig. 4G). In the same animals, IC disorganization and nuclei scattering phenotype was more severe compared to that in animals with *pelo* knockdown alone (Fig. 4C,H). Furthermore, there were about 10.6 ICs per testis in *Hbs1* transheterozygotes (*Hbs1*^*1*^ over *Hbs1*^*172*^), and about 12.6 ICs per testis in *pelo*^*PB60*^ heterozygotes (*pelo*^*PB60*^−/+), but IC number dropped significantly to an average of 4.2 when one copy of *pelo* was removed, and both the IC and nuclei were severely disorganized (Fig. 4D–H). These observations indicate that *pelo* and *Hbs1* genetically interact with each other to promote spermatid individualization during spermatogenesis.

### Hbs1 co-localizes with Pelo in germ cell cytoplasm and they physically interact with each other

**Figure 5:**
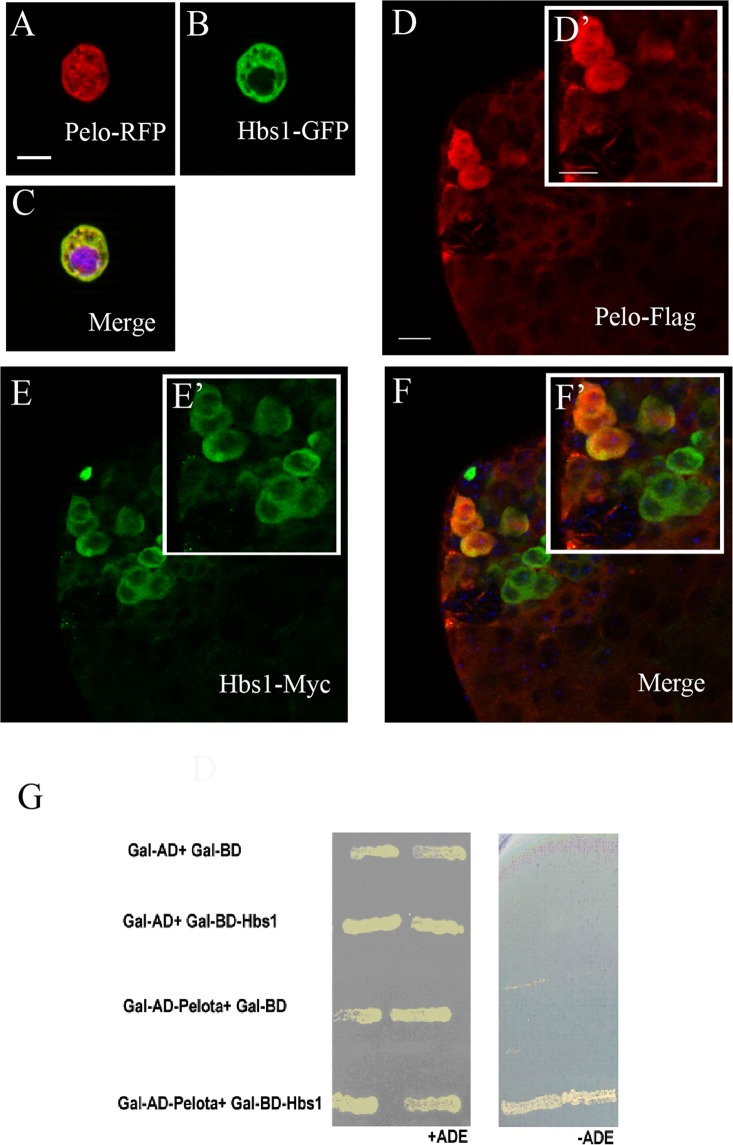
Pelo physically interacts with Hbs1. (**A**–**C**) Co-expression of Pelo (**A**, Pelo-RFP, red) and Hbs1 (**B**, Hbs1-GFP, green) in *Drosophila* S2 cells, where those two proteins were co-localized in the cytoplasm (**C**: merged image with DAPI in blue). (**D**–**F**) UASp-Pelo-flag (**D**, red) and UASp-Hbs1-myc (**E**, green) were both expressed in the cytoplasm of testicular germ cells when they were driven by nosGal4. Note that they could co-localize with each other in the germline cells (**F**). (**G**) Pelo interacted with Hbs1 in a yeast two hybrid assay. Scale bar represents 10 μm in **A**–**C** and 15 μm in **D**–**F**.

Next, we asked whether Pelo and Hbs1 could form a protein complex in *Drosophila*. When Pelo tagged with RFP and Hbs1 tagged with GFP at their C terminal regions were co-expressed in *Drosophila* S2 cells, they predominantly co-localized in the cytoplasm (Fig. 5A–C). Interestingly, unlike Hbs1-GFP, a significant fraction of Pelo-RFP was also localized to the nucleus (Fig. 5A). We also constructed UAS-pelo-FLAG and UAS-Hbs1-myc transgenes, and co-expressed them in the male germline. Both protein products were found to co-localize with each other in the cytoplasm of germ cells (Fig. 5D–F and insets D’–F’). Additionally, Hbs1 and Pelo exhibited strong interaction with each other in a yeast-two-hybrid assay (Fig. 5G). In summary, these data suggest that Hbs1 and Pelo form a protein complex, and along with the evidence for a genetic interaction during spermiogenesis, they likely function in a same protein complex to regulate late spermatogenesis in *Drosophila*.

### The putative Hbs1-binding motif is important for Pelo function *in vivo*

**Figure 6:**
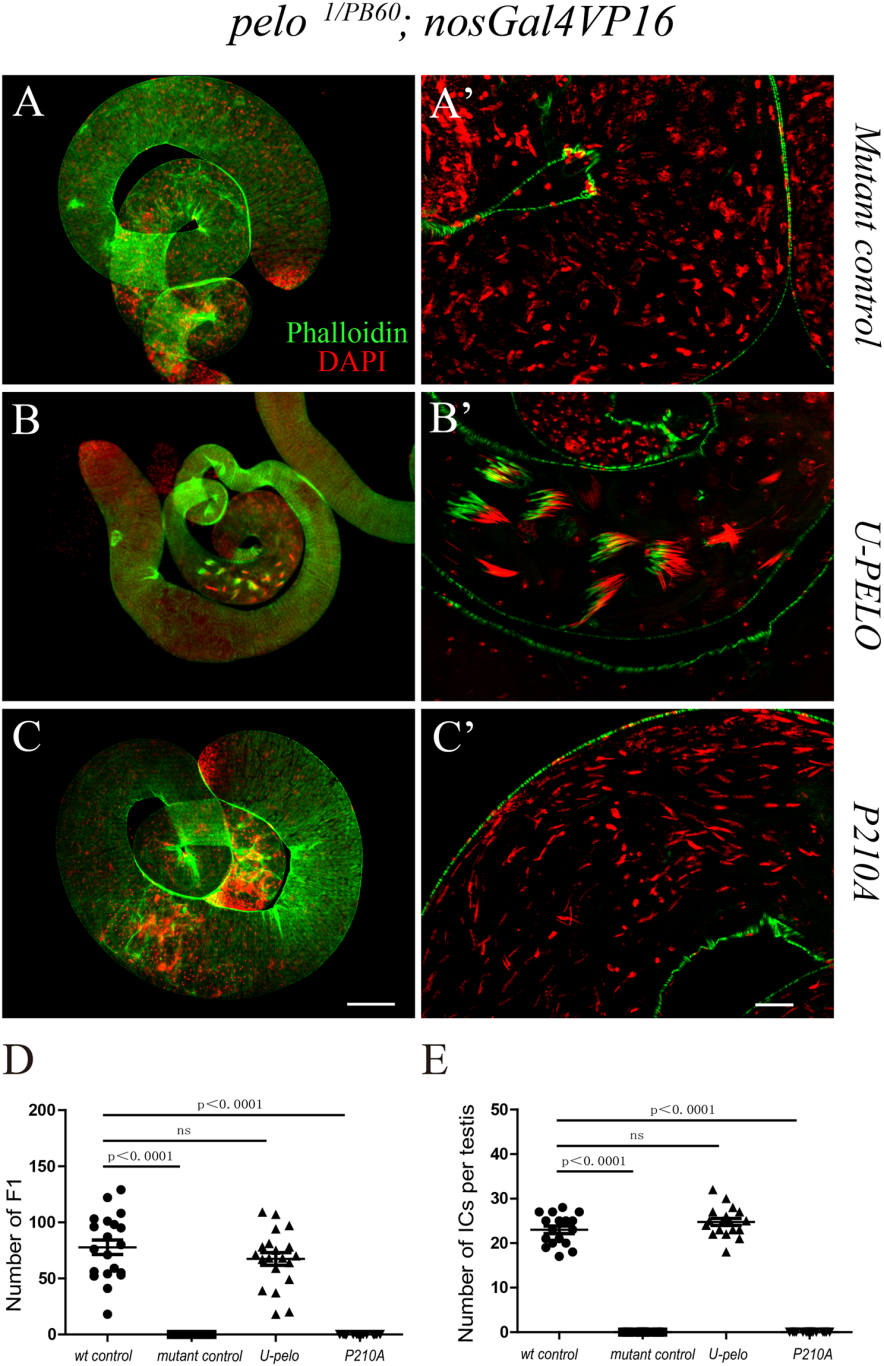
The putative Hbs1-binding motif of Pelo is important for its function. The effect of germline expression of different transgenes on rescuing spermatid defects in *pelo*^*1/PB60*^ testis. (**A**,**A’**) The mutant control testis without transgene expression. (**B**,**B’**) Transgene expression of Pelo (WT). Note that ICs were properly formed. (**C**,**C’**) Transgene expression of Pelo (P210A). Note that ICs failed to form. ICs were visualized by phalloidin staining (in green) and spermatid nuclei were recognized by DAPI (in red). Scale bars represent 100 μm in (**C**), and 20 μm in (**C’**). (**D**,**E**) Quantitative analysis: transgene expression of wild type pelo could fully rescue the IC loss (**E**) and male sterility (**D**) caused by pelo mutant phenotype, but P210A could not.

To test the functional importance of the interaction between Pelo and Hbs1 during spermatogenesis, we asked whether specifically disrupting the interaction between Pelo and Hbs1 could disrupt their function during spermatogenesis. Structural and biochemical data have shown that the conserved PGF motif in the central domain of Dom34 interacts with a conserved RDF motif in the GTPase domain of Hbs1, and the PGF motif is required for both the interaction of Pelo with Hbs1 and the no-go-decay activity^8,18^. We therefore used a transgene of Pelo carrying Pro210 to Ala (P210A) mutation that is supposed to disrupt the Pelo-Hbs1 complex function possibly by altering the allostatic configuration of the interaction interface^29^, and tested its ability in rescuing spermatogenesis defects caused by the loss of Pelo using a germline-specific driver nosGAL4 (Fig. S3A)^38^. As described earlier, in wild type testes, the nuclei bundles and ICs were detected by DAPI and phalloidin staining, respectively (Fig. 3A–C). In strong *pelo* mutants (*pelo*^*1/PB60*^), cell cycle was arrested at early stages of meiosis and there was no elongated spermatid nuclei bundles and therefore no IC formed in these animals (Fig. 6A–A’,E). As a control, the spermatogenesis defects and the sterility were fully rescued by a wild type transgene Pelo (WT) (Fig. 6B–B’, D,E). However, the defects largely remained unrescued by Pelo (P210A), as ICs could not be formed, although spermatid nuclei seemed to get elongated to some extent (Fig. 6C–C’,D,E). Interestingly, Pelo (P210A) was able to fully rescue the GSC loss phenotype in the ovary (data not shown), which is consistent with the observation that Hbs1 is not required for GSC maintenance, as shown below. Pelo (P210A) partially rescued the GSC loss phenotype in the testis while the wild type Pelo could fully rescue it (Fig. S3), indicating the possible participation of other co-factors that bind to Pelo to maintain the GSCs in the testis. These data suggest that the putative Hbs1-binding motif is required for Pelo function during late spermatogenesis, further supporting the notion that Pelo and Hbs1 from a complex to regulate late spermatogenesis.

### *Hbs1* is not required for ovarian/testicular GSC function

Previous studies demonstrate that *pelo* is required for GSC self-renewal and oocyte development during oogenesis^28^. We therefore asked whether *Hbs1* is also required for the self-renewal of GSCs. Interestingly, no defect was observed in either GSC maintenance or germline cyst differentiation in *Hbs1* mutant females (data not shown). Similar to its role in ovarian GSCs, we found that *pelo* was also important for the maintenance of testicular GSCs (Fig. S4A–F). However, *Hbs1* mutants showed normal GSC number and proper early cyst differentiation (Fig. S4G, J–M), even in aged males (40 days old). Additionally, the gradual GSC loss phenotype caused by *pelo* mutation was not further enhanced by the loss of *Hbs1* (Fig. S4H–J). These data together suggest that *Hbs1* is not required for the maintenance of GSCs, implying that the stem cell function of Pelo is independent of Hbs1.

## Discussion

### Hbs1 regulates spermatogenesis in collaboration with Pelo

Here we demonstrate a novel and specific function of Hbs1 in spermatogenesis in *Drosophila*. Loss of function of *Hbs1* causes defects in meiotic cell division and spermatid differentiation during spermatogenesis, leading to male infertility with no apparent defect in viability, and this revealed a male-specific germline function of Hbs1.

Our results also suggest that Hbs1 may collaborate with Pelo to regulate multiple processes during spermatogenesis in *Drosophila*, which is supported by several observations. First, Hbs1 physically interacts with Pelo in a yeast-two-hybrid assay. Hbs1 also co-localizes with Pelo in the cytoplasm of S2 cells and germline cells. Second, Hbs1 and Pelo genetically interact with each other during spermatid individualization, as phenotypes caused by partial loss of function in one can be enhanced by the loss of function of the other. The phenotypes produced by *Hbs1* or *pelo* mutations could not be rescued by the UAS-pelo and UAS-Hbs1 respectively (data not shown), which excludes the possibility that their function can compensate for each other. Finally, the putative Hbs1-binding motif-mutant version of Pelo cannot substitute the wild type Pelo for spermatogenesis, further supporting the idea that Pelo and Hbs1 form a protein complex to function during spermatogenesis. Our results, together with the previously reported results that yeast as well as mammalian homologues of Hbs1 and Pelo interact with each other^6,14,15,24^, collectively support that the physical interaction between Hbs1 and Pelo are evolutionary conserved from yeast to mammals.

### Pelo-Hbs1 complex may regulate translation during spermatogenesis

Hbs1-Pelo complex likely regulates spermatogenesis through translational regulation. Hbs1 belongs to a protein family known to be involved in several steps of translational processes, including translational elongation, release, and decay of nonfunctional mRNAs^39,40^. Also, increased expression of Hbs1 in yeast could suppress the growth defect in strains deficient for *SSB1/2*, which encode molecular chaperones that are associated with nascent polypeptides and function to facilitate transportation of newly synthesized peptides to the cytosol^5^. In addition, yeast *Hbs1* mutants show defective protein synthesis upon limiting translation initiation^6^. Consistently, in partnership with Dom34/Pelo, Hbs1 has been demonstrated to be involved in no-go and no-stop mRNA decay^41,42^. In this study, expression of a tagged Hbs1 in the male gonad reveals that Hbs1 is predominantly localized in the cytoplasm of germline cells, consistent with a role of Hbs1 in translational regulation. Dom34/Pelo and Hbs1 have been shown to have a role in degrading aberrant 18S rRNAs. Nonfunctional rRNAs and mRNAs to be removed by no-go decay are both localized within P bodies, places where mRNA decay is conducted^43^. Therefore, Pelo-Hbs1 could remove both nonfunctional rRNAs and aberrant mRNAs for proper spermatogenesis. In mammalian cells, the Pelo-Hbs1complex along with ABCE1 is important for recycling vacant 80S ribosomes^12^, it is therefore possible that Pelo-Hbs1 are important for ribosome recycling to ensure proper spermatogenesis.

Despite our finding to show that Hbs1 and Pelo function together during late spermatogenesis, our results also indicate Hbs1-independent functions of Pelo in maintaining both male and female GSCs and in early spermatogenesis. Hbs1 is dispensable for GSC maintenance and mutants show relatively weaker phenotype in male meiosis compared to that of *Pelo* mutants^3,28^. This is similar to the situation in yeast, in which *Dom34/Pelo* mutants display stronger and more profound phenotypes, such as defective sporulation and decreased polyribosomes, whereas *Hbs1* mutants show no obvious phenotype^4,6^. Moreover, Hbs1 seems to play a minor role in no-go decay compared to the critical requirement of Dom34/Pelo^7^. It is possible that there are other redundant G proteins that can functionally substitute Hbs1 or interact with Pelo at different stages together with Hbs1 during spermatogenesis, and Pelo may be a key player in the complex, while Hbs1 may only have a supportive role.

### Translational regulation during spermatogenesis: multiple levels of control

Translational regulation plays crucial roles in biological processes in which transcription is largely absent and developmental events are obligatory to be carried out. It is known that transcription is nearly absent at postmeiotic stage during spermatogenesis in *Drosophila*, while a short period of transcription exists postmeiotically in mammals^1,44,45^. So translational regulation is critical in regulating gene expression during the postmeiotic stage, where germ cells undergo dramatic remodeling while transcription is shut down in spermiogensis^46–48^. In those circumstances, many mRNAs are produced and translationally repressed in spermatocytes until the encoded proteins are required for later spermatogenesis, such as *don juan* and *don juan like*, both of which are initially transcribed in primary spermatocytes but translationally repressed until spermatid elongation^47,49^. Translational activation is also required for the sequential gene expression in later development in spermatogenesis. Boule, *Drosophila* orthologue of the vertebrate Y-linked *Deleted in Azoospermia-like* (*DAZl*) promotes the efficient translation of *twine* for meiotic entry^50^. The general translation system, which includes translation initiation, elongation, and termination, also contributes to the expression of different genes at different stages of germline development. eIF4G and eIF4G2, paralogues of the translation initiation complex component eIF4G, are required for meiotic progression and spermatid differentiation in *Drosophila* by controlling temporal expression of core cell cycle regulators, implicating that alternate translation initiation machinery may be responsible for gene expression and germline development in spermatogenesis^51–53^. Our results here suggest another level of translational regulation during spermatogenesis mediated by RNA surveillance factors. The *Drosophila* Pelo-Hbs1 complex, which has been demonstrated to be required for no-go decay in cultured *Drosophila* cells and transposon silencing in female germline^13,29^, could be involved in degrading poisonous/defective mRNAs such as transposon transcripts, rRNAs and/or facilitating ribosome recycling that would otherwise lead to defects in meiosis and spermatid differentiation. Conditional depletion of *Pelo* in postnatal mice causes progressive spermatogonial stem cell loss and sterility, although later spermatogenesis appears normal before germline exhaustion^27^. It remains possible that the perdurance of gene product follow gene knock-out in stem cells may be sufficient to allow later spermatogenesis. Therefore it would be worthwhile to re-evaluate the potential function of Pelo-Hbs1 complex in mammalian gametogenesis by germline depletion of Hbs1.

## Materials and Methods

### *Drosophila* strains

All flies were raised at 25 °C and cultured on standard media, unless otherwise stated. The following fly stocks were used in this study include: *pelo*^*1*^, *pelo*^*PB60*^, *pelo*^*PA13*^; *Hbs1*^*1*^, *Hbs1*^*48*^ and *Hbs1*^*172*^ (this study, see in the text for molecular lesions); *Pelo (P210A)*^29^ *and* nosGal4VP16^38^. BamGAL4VP16 (a gift from Dahua Chen)^54^. Vasa-GAL4 (a gift from Zhaohui Wang)^55^, and *pelo* RNAi lines were from VDRC (v34770; v34771)^56^.

### Generation and characterization of new *pelo* alleles

*pelo*^*PB60*^ was caused by a 107 bp deletion in the second exon, and the predicted protein product is a peptide with 77 amino acids (compared to the full length with 395 amino acids). Flies homozygous for *pelo*^*PB60*^ showed similar GSC loss phenotype during oogenesis and meiosis defect during spermatogenesis to transheterozyotes (*pelo*^*PB60*^ in trans to a deficiency *Df(2 L)Exel6024* in which the entire *pelo* locus is deleted). *pelo*^*PB60*^ homozygous testes also displayed similar severity of meiosis defects to that of *pelo*^*1*^ homozygous mutants, suggesting that both *pelo*^*PB60*^ and *pelo*^*1*^ alleles can be considered as strong loss of function or genetic null alleles. *pelo*^*PA13*^ was resulted from a 557 bp insertion left by the original KG6646 P element, and homozygous mutant cysts from testes squash displayed meiosis arrest either before the first or the second meiotic cell division. Based on the severity of defects in female GSC maintenance and male meiosis, we considered *pelo*^*PA13*^as a hypomorphic allele.

### Remobilization of P element to generate new alleles

The pelo^KG6646^ chromosome was brought together with a transposase source by crossing pelo^KG6646^/Cyo flies to CyO, PBac{w[+mC] = Delta2-3.Exel}2/amos[Tft] flies which carried transposase on the second chromosome (Bloomington stock number:8201). Then the flies with pelo^KG6646^/CyO, PBac{w[+mC] = Delta2-3.Exel}2 were crossed to those with CyO/Sco. In the next generation, pelo[]/CyO flies without CyO, PBac{w[+mC] = Delta2-3.Exel}2 chromosome were selected to establish excised lines. *Hbs1* alleles were established in a similar way using Hbs1^EY09557^.

### Generation of transgenic flies and expressing vectors

For the genomic rescue experiment, the genomic region of *Hbs1* together with 1X Flag coding region at the C-terminal end was amplified by PCR using KOD polymerase (Takara Bio, Otsu) and cloned into Spe I and Not I sites of pCasPeR 4. The following primers were used: 5′ GGACTAGTCC TGAAGCAAAT CAGAACAGTC 3′ and 5′ TTGCGGCCGCAA CTACTTGTCATCGTCATCCTTGTAATC GCG GAT CTT GGT GAC CAT T 3′. To generate epitope-tagged transgenes, ORFs of both *pelo* and *Hbs1* were first cloned into pENTRY/D-TOPO vector and then into pPWF and pPWM vectors respectively through the Gateway reaction to make *UASP-Pelo-FLAG* and *UASP-Hbs1-Myc* (The *Drosophila* gateway vector collection developed by Terrence Murphy: http://emb.carnegiescience.edu/labs/murphy/Gateway%20vectors.html). To generate *pelo* carrying Pro210 to Ala (P210A) mutation, *pelo* was cloned from Canton-S ovarian cDNA library and ligated into pEasy-blunt Simple vector (TransGen Biotech). Single amino acid substitution was generated by Easy Mutagenesis System (TransGen Biotech) according to the manufacturer’s instruction. Full length *pelo* coding sequence was amplified by PCR with primers 5′-GGTACCATGAAGCTGCTGGGCAAATA-3′ and 5′-TCTAGACTAGTCGCTATCGCTATCTGCC-3′. The PCR fragment was restriction digested with KpnI and XbaI, and subsequently cloned into pUASP. All the plasmid DNA was injected for transformation via standard procedures.

To generate Pelo-RFP and Hbs1-GFP constructs for expression in *Drosophila* S2 cells, ORFs of *Pelo* and *Hbs1* were cloned into pAWG and pAWR vectors using the Gateway kit. Constructs were transfected into *Drosophila* S2 cells using the standard calcium phosphate transfection protocol, and the expression pattern of Pelo-RFP and Hbs1-GFP was examined 4 days after the initial transfection.

### RNA isolation and qPCR assays

Total RNA from 50–60 testis was extracted by RNAiso Plus reagent(Takara,9109) and Direct-zol RNA Miniprep kit(Epigenetics, R2050) with DNase treatment. Complementary DNA(cDNA) was synthesized by 5X All-In-One RT MasterMix kit(abm,G490). RT-qPCR was performed in three duplicates using SYBR Select Master Mix(Thermo Fisher,4472908) on ABI PRISM 7500 fast Real-time PCR System(Applied Biosystems). Endogenous *Actin5c* was used for normalization. Fold changes for mRNA levels were calculated using ΔΔ*C*_t_ method^57^. The primers used are listed in Table S1.

### Fertility assay

Fertility assay was performed as previously described^58^. Each male was put together with two *w*^1118^ virgin females in one food vail in twenty duplicates. The flies were raised at 25 °C and separated from each other after 5 days. The offspring were examined within 2 weeks.

### Analysis of testis content

Newly enclosed males were collected and manually dissected for testes in Testis Buffer (183 mM KCl, 47 mM NaCl, 10 mM Tris-HCl, 1 mM EDTA, 1 mM PMSA). Testes were subsequently transferred onto a coverslip containing a 2-ul drop of Testis buffer. Testes were then opened using forceps, squashed and examined by phase contrast using a Zeiss Axio microscope.

### Immunohistochemistry

Fly testes were dissected in PBS medium, fixed in 4% paraformaldehyde for 10 min at room temperature, and then washed with PBT (PBS containing 0.1% Triton X-100). For DAPI (49,6-diamidino-2-phenylindole) staining, samples were incubated with 1 mg/mL DAPI in PBT for 6 min, and the reaction was stopped by washing samples with PBT. For phalloidin staining, samples were incubated with rodamine-phalloidin (Invitrogen; 1:10) at 37 °C for 2 hours and subsequently washed with PBT twice. For antibody staining, samples were fixed first and washed three times with PBST. After blocking with 5% normal goat serum in PBT, primary and secondary antibodies were then added according to standard procedures. Antibodies used in this study include anti-α-spectrin antibody (3A9, Developmental Studies of Hybridoma Bank (DSHB), 1:50 dilution), anti-DE-Cadherin (DCAD2, DSHB, 1:50 dilution), anti-Vasa (a gift from Y. N. Jan, 1:100 dilution), anti-FLAG (F1804, Sigma), anti-Myc (DSHB, 1:50), anti-α-tublin (Bio-Rad, MCA78G,1:200), Alexa568-conjugated goat anti-mouse and Alexa 488-conjugated goat anti-rabbit secondary antibodies (Molecular Probes, Carlsbad, CA, USA, 1: 300 dilution). Samples were then mounted in 70% glycerol in 1 × PBS. Images were captured by either a Zeiss Imager Z1 equipped with an ApoTome system or a Zeiss Meta 510 confocal microscope. All images were processed in Adobe Photoshop and Illustrator.

### Yeast-two hybrid

ORF of Pelota was cloned into NdeI and EcoRI sites of pGADT7 AD Vector. The primers used were 5′ CCCATATGAAGCTGCTGGGCAAATACGT 3′, and 5′ CCGAATTCTAGTCGCTATCGCTATCT 3′. ORF of Hbs1 was cloned into Nde I and BamH I sites of pGBKT7 DNA-BD vector, and primers were 5′ CCCATATGTCGCGGCACAGGATAGT 3′ and 5′ CCGGATCCTAGCGGATCTTGGTGACCATT 3′. The yeast two hybrid assay was based on the MATCHMAKER kit (Clontech). pGBKT7-p53 with the Gal4 DNA-BD fused with murine p53 and pGADT7-T with the Gal4 AD fused with SV40 large T-antigen were used as positive control.

## Supplementary information

Supplementary info

## Data Availability

No datasets were generated or analyzed in the current study.
