# Supplementary material for: Pelota-interacting G protein Hbs1 is required for spermatogenesis in Drosophila
Source: Sci Rep. 2019 Mar 1;9:3226. doi: 10.1038/s41598-019-39530-6 (PMC6397311; doi:10.1038/s41598-019-39530-6)
Supplement: Supplementary file 1 — Supplementary info [file 41598_2019_39530_MOESM1_ESM.pdf]

Pelota-interacting G protein Hbs1 is required for spermatogenesis in *Drosophila*

Zhaohui Li, Fu Yang, Yang Xuan, Rongwen Xi, Rui Zhao

Supplementary Information

Supplementary Figure Legends

Figure S1. Sequence alignment among Hbs1 orthologs across different species. gi|6322937|ref|NP_013010.1| Hbs1 [Saccharomyces cerevisiae]; gi|30683251|ref|NP_196625.2| putative EF-1-alpha [Arabidopsis thaliana]; gi|115532067|ref|NP_001021556.2| K07A12.4b [Caenorhabditis elegans]; gi|45550900|ref|NP_652729.2| HBS1 CG1898-PA [Drosophila melanogaster]; gi|148223485|ref|NP_001085851.1| MGC80911 protein [Xenopus laevis]; gi|118088523|ref|XP_001234091.1| PREDICTED: HBS1-like [Gallus gallus]; gi|41054437|ref|NP_955970.1| Hbs1-like [Danio rerio]; gi|110611222|ref|NP_062676.2| Hbs1-like isoform 1 [Mus musculus]; gi|5729864|ref|NP_006611.1| HBS1-like [Homo sapiens].
Identical amino acids are highlighted by black boxes, and similar amino acids are highlighted by grey boxes.

Figure S2. Pelo is required for both meiosis and spermatid individualization during spermatogenesis. (A-F) Phase-contrast photographs analyzing the content of unfixed testis of wild type and *pelo* mutant flies (n=2). Onion stage spermatids with dark nebenkern (arrow) and light nuclei (arrow head) of similar size were detected in wild type testis (A-B). *pelo*$^{PB60-/-}$ and *pelo*^{PA13} mutants showed defects of meiosis, with abnormally large spermatids containing 4N (arrow) or 2N (arrow head) chromosomal content with large nebenkern (hollow arrow) (C-F). (G) ICs (marked by Phalloidin in red) could not be detected and the nuclei are scattered (marked by dapi in blue, arrow) in *pelo*$^{PB60-/-}$ mutant testis. (H-I)There are intact ICs (marked by Phalloidin in red, arrow) both at the distal end and the apical end of the testis in the nos-Gal4 driver alone(H, a genetic control for Fig4A-G), There was also no significant difference between the number of ICs in *nos-Gal4* driver lines and that in *Hbs*$^{1/+}$ lines(I). Mean ± SEM. *n*=20. ns, no significance. *t test*. In A-F, scale bars = 10μm. In G-H, scale bars = 50μm.

Figure S3. The Hbs1-binding motif of Pelo is important for its function.

(A) Quantitative RT-PCR analysis of the relative *pelo* expression. In the mutant fly, the expression of *pelo* decreased to almost half of the wild type, and both transgenes are correctly expressed. (B) P210A could only partially rescue the GSCs loss. wild type is *w1118*, the mutant is *pelo*$^{1/PB60}$*; nosGal4VP16***,** U-pelo is *pelo*$^{1/PB60}$*; nosGal4VP16;U-pelo* and P210A is *pelo*$^{1/PB60}$*; nosGal4VP16;U-P210A*.

Figure S4. *pelo*, but not *Hbs1*, is required for male GSC maintenance. (A) A testis genotyped *pelo*^{PA13}*/Cyo* contains 8-10 GSCs per hub (asterisk, marked by E-cadherin, red) on average at day 7. (B) The GSC (marked by cells stained with vasa (green) in close contact with the hub) number was significantly decreased in flies that were homozygous for *pelo*^{PA13}. (C) GSCs continued to decrease towards day 15. (D) On average, 8-10 GSCs could be found at the hub of a *pelo*^{PB60}*/Cyo* testis at day 7. (E) About 5 GSCs left in *pelo*$^{PB60-/-}$ mutant testis at day 7. (F) Only 2-3 GSCs existed in those *pelo*$^{PB60-/-}$ mutant testis at day 15. (G) A *Hbs1*$^{1-/-}$ testis at day 20 was shown with around 8-9 GSCs surrounding the hub. (H-J) Double mutant testis genotyped either for *pelo*$^{PA13-/-}$; *Hbs1*$^{1-/-}$ (H) or *pelo*$^{PB60-/-}$; *Hbs1*$^{1-/-}$ (I) showed neither suppression nor enhancement of the GSC loss phenotype compared to the *pelo* single mutants along (day 15). The averaged GSC numbers per hub under different genetic conditions are illustrated in J (7D, 15D and 20D are the abbreviation for 7days, 15days and 20days, respectively). (K-M') *Hbs1*$^{1-/-}$ showed no effect on spermatogonial (Bam-GFP positive cells. K' and M' are enlarged figures of K and M, respectively. DAPI is in blue in from A-I,K-L. GSCs and Bam-GFP+ cells are outlined by dotted lines. In A-I, scale bars = 20μm. In K-L, scale bars = 50 μm.

Supplementary Figures

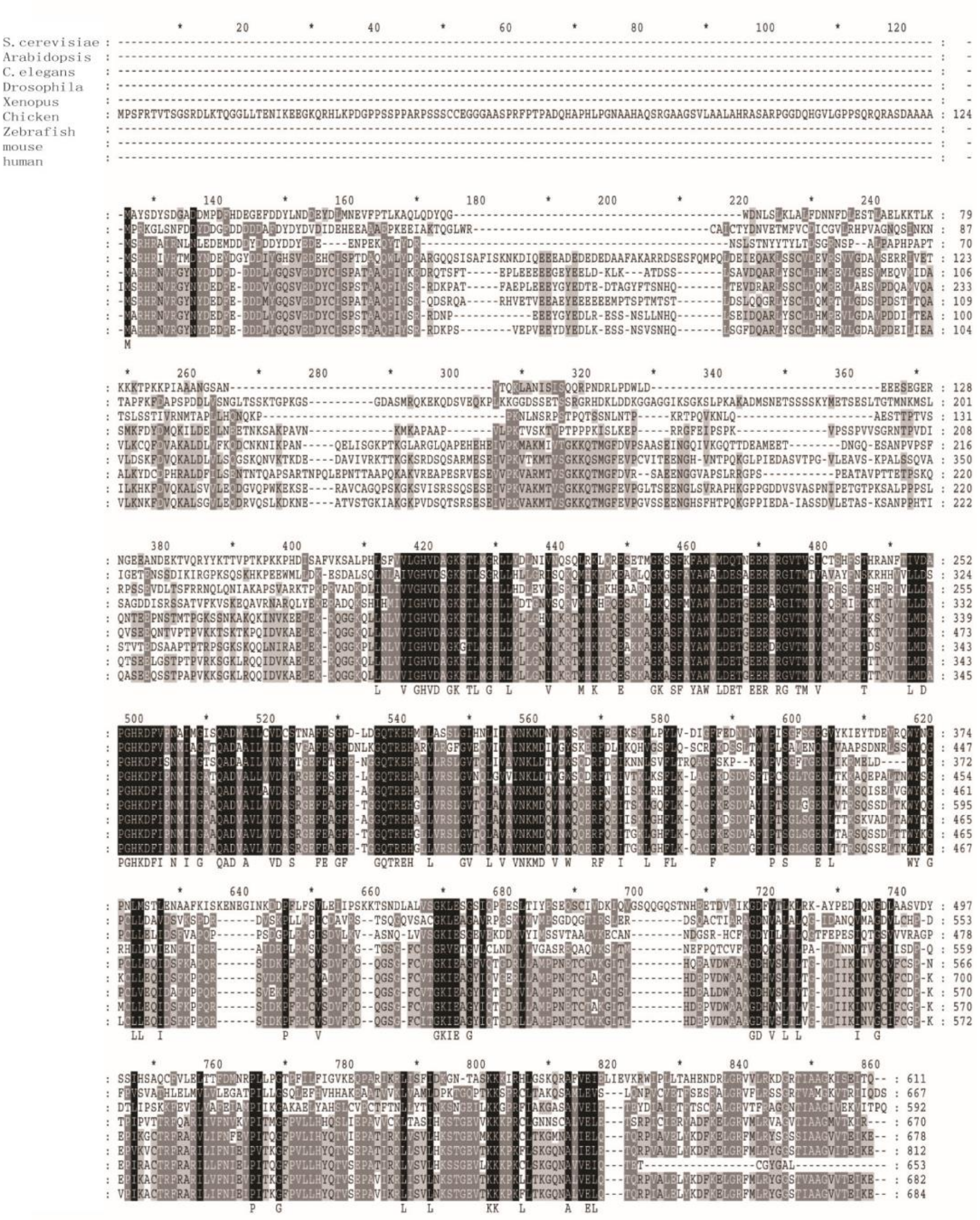

Figure S1

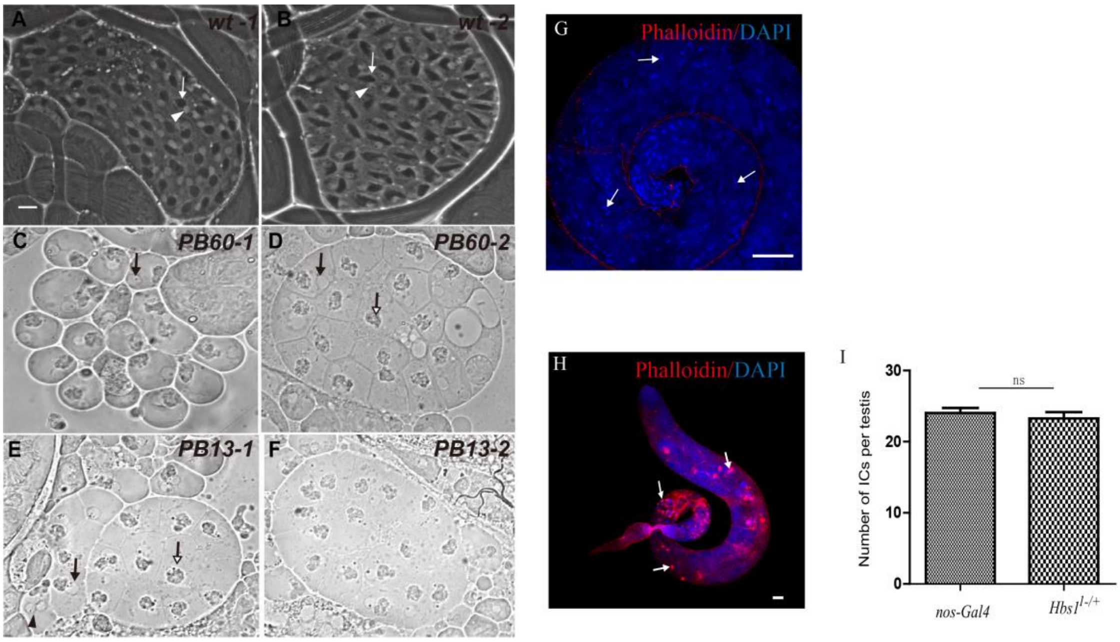
A
wt -1
B
wt -2
C
PB60-1
D
PB60-2
E
PB13-1
F
PB13-2
G
Phalloidin/DAPI
H
Phalloidin/DAPI
I
Number of ICs per testis
30
20
10
0
ns
nos-Gal4
Hbs1[1-/+]

Figure S2

A

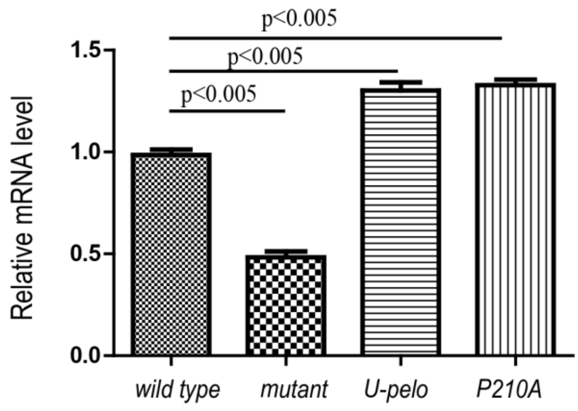

p<0.005
p<0.005
p<0.005
Relative mRNA level
1.5
1.0
0.5
0.0
wild type
mutant
U-pelo
P210A

B

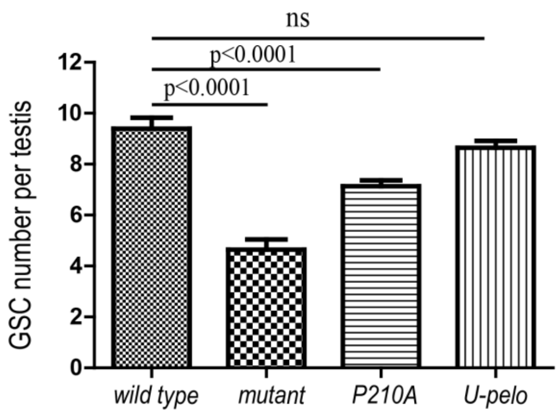

ns
p<0.0001
p<0.0001
GSC number per testis
12
10
8
6
4
2
0
wild type
mutant
P210A
U-pelo

Figure S3

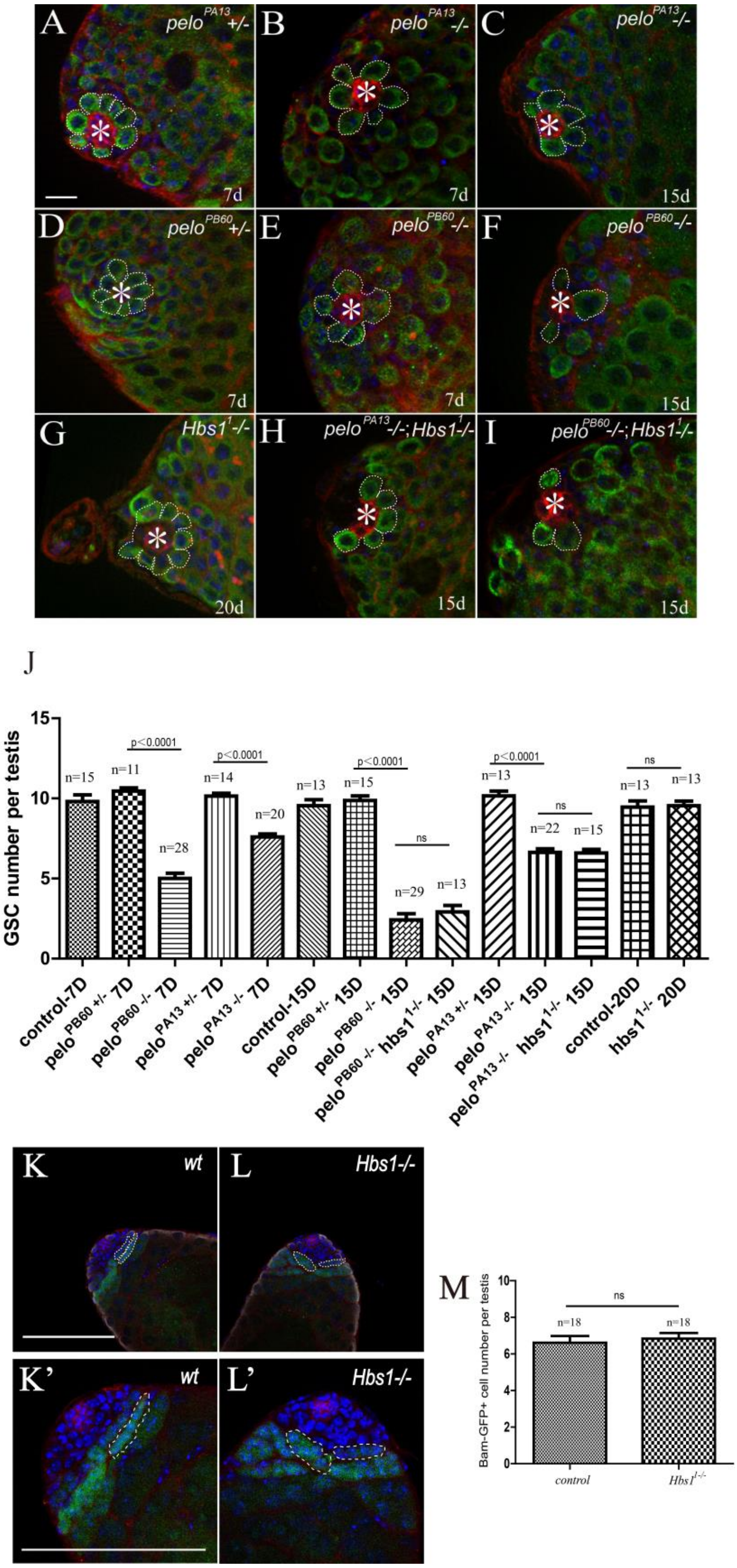
A
pelo PA13 +/-
7d
B
pelo PA13 -/-
7d
C
pelo PA13 -/-
15d
D
pelo PB60 +/-
7d
E
pelo PB60 -/-
7d
F
pelo PB60 -/-
15d
G
Hbs1 1 -/-
20d
H
pelo PA13 -/-; Hbs1 1 -/-
15d
I
pelo PB60 -/-; Hbs1 1 -/-
15d
J
GSC number per testis
15
10
5
0
p<0.0001
ns
n=15
n=11
n=28
n=14
n=20
n=13
n=15
n=29
n=13
n=13
n=22
n=15
n=13
n=13
control-7D
pelo PB60 +/- 7D
pelo PB60 -/- 7D
pelo PA13 +/- 7D
pelo PA13 -/- 7D
control-15D
pelo PB60 +/- 15D
pelo PB60 -/- 15D
pelo PB60 -/- hbs1 1-/- 15D
pelo PA13 +/- 15D
pelo PA13 -/- 15D
pelo PA13 -/- hbs1 1-/- 15D
control-20D
hbs1 1-/- 20D
K
wt
L
Hbs1-/-
K'
wt
L'
Hbs1-/-
M
Bam-GFP+ cell number per testis
10
8
6
4
2
0
ns
n=18
n=18
control
Hbs1 1-/-

Figure S4
